# Non-destructive Evaluation of the Quality Characteristics of Pomegranate Kernel Oil by Fourier Transform Near-Infrared and Mid-Infrared Spectroscopy

**DOI:** 10.3389/fpls.2022.867555

**Published:** 2022-07-07

**Authors:** Emmanuel E. Okere, Ebrahiema Arendse, Helene Nieuwoudt, Willem J. Perold, Umezuruike Linus Opara

**Affiliations:** ^1^SARChI Postharvest Technology Research Laboratory, Africa Institute for Postharvest Technology, Faculty of AgriSciences, Stellenbosch University, Stellenbosch, South Africa; ^2^Department of Electrical and Electronic Engineering, Stellenbosch University, Stellenbosch, South Africa; ^3^Department of Viticulture and Oenology, Institute for Wine Biotechnology, Stellenbosch University, Stellenbosch, South Africa; ^4^UNESCO International Centre for Biotechnology, Nsukka, Enugu State, Nigeria

**Keywords:** *Punica granatum* L., oil quality, partial least squares regression, discriminant analysis, infrared spectroscopy

## Abstract

The pomegranate kernel oil has gained global awareness due to the health benefits associated with its consumption; these benefits have been attributed to its unique fatty acid composition. For quality control of edible fats and oils, various analytical and calorimetric methods are often used, however, these methods are expensive, labor-intensive, and often require specialized sample preparation making them impractical on a commercial scale. Therefore, objective, rapid, accurate, and cost-effective methods are required. In this study, Fourier transformed near-infrared (FT-NIR) and mid-infrared (FT-MIR) spectroscopy as a fast non-destructive technique was investigated and compared to qualitatively and quantitatively predict the quality attributes of pomegranate kernel oil (cv. Wonderful, Acco, Herskawitz). For qualitative analysis, principal component analysis (PCA) and orthogonal partial least squares discriminant analysis (OPLS-DA) was applied. Based on OPLS-DA, FT-MIR spectroscopy resulted in 100% discrimination between oil samples extracted from different cultivars. For quantitative analysis, partial least squares regression was used for model development over the NIR region of 7,498–940 and 6,102–5,774 cm^−1^ and provided the best prediction statistics for total carotenoid content (*R*^2^, coefficient of determination; RMSEP, root mean square error of prediction; RPD, residual prediction deviation; *R*^2^ = 0.843, RMSEP = 0.019 g β-carotene/kg, RPD = 2.28). In the MIR region of 3,996–1,118 cm^−1^, models developed using FT-MIR spectroscopy gave the best prediction statistics for peroxide value (*R*^2^ = 0.919, RMSEP = 1.05 meq, RPD = 3.54) and refractive index (*R*^2^ = 0.912, RMSEP = 0.0002, RPD = 3.43). These results demonstrate the potential of infrared spectroscopy combined with chemometric analysis for rapid screening of pomegranate oil quality attributes.

## Introduction

Pomegranate and its co-products have gained traction in research and application for their nutraceutical and medicinal properties (Seeram et al., 2006; Opara et al., 2009). Pomegranate fruit can be divided into two fractions: edible and non-edible fractions. The edible portion contains arils and each aril contains a kernel (woody portion; O’Grady et al., 2014; Arendse et al., 2016). Pomegranate oil is derived from the kernel of the fruit and studies over the years have reported that the oil derived from the kernels have radical scavenging activity, anti-inflammatory, anti-tumoral and anti-diabetic properties (Lansky et al., 2005; Lansky and Newman, 2007; Jing et al., 2012; Fernandes et al., 2015; De Melo et al., 2016). These properties have been linked to its unique phenolic and fatty acid composition (Seeram et al., 2006; Khoddami et al., 2014; Fernandes et al., 2015). Pomegranate oil carries a higher premium compared to other oils such as olive and avocado oil, the premium may be due to its unique fatty acid composition only found within pomegranates as well as its high phytochemical composition. Thus pomegranate oil, among others is highly susceptible to adulteration with cheaper alternatives (Uncu et al., 2020).

To evaluate the chemical constituents in oil products, standard analytical methods such as high-performance liquid chromatography and various colorimetric methods are used (Dieffenbacher and Pocklington, 1991; Aluyor et al., 2009). These methods are used to provide precise and accurate measurements of quality attributes. However, their approach is often time-consuming, expensive, and not always practical for large-scale commercial applications as it involves the use of trained sensory panelists or individuals. These drawbacks have promoted research interest in developing objective and non-invasive techniques for faster and less expensive assessment of oil quality attributes.

Due to its rapid, accurate, simple, and cost-effective way to evaluate chemical constituents, infrared (IR) spectroscopy in combination with chemometrics is one of the widely used non-destructive tools used by the food and beverage industry for quality testing and analysis (Sinelli et al., 2010; Becker and Yu, 2013; Shi and Yu, 2017). IR spectroscopy is appropriate for predicting compounds containing polar functional groups such as –OH, C–O, and N–H. In the agricultural industry, IR spectroscopy in the near-infrared (NIR, 12,500–4,000 cm^−1^) and the mid-infrared (MIR, 4,000–400 cm^−1^) spectral region has been applied as a non-destructive analytical tool. Fourier transform infrared spectroscopy (FT-IR) uses the mathematical process (Fourier transform) to translate the raw data (interferogram) into the actual spectrum. FT-IR spectrometers have recently replaced dispersive instruments, due to their superior speed and sensitivity. FT-IR spectrometers have several prominent advantages over dispersive IR spectrometers. A better signal-to-noise ratio of the spectrum compared to the previous generation infrared spectrometers. FT-IR spectrometers have a higher wavenumber accuracy and low error range (±0.01 cm^−1^). Their scan time is short (approximately 1 s) and has a high resolution (0.1–0.005 cm^−1^; Hsu, 1997).

In combination with chemometric tools, both Fourier transform near-infrared spectroscopy (FT-NIRs) and Fourier transform mid-infrared spectroscopy (FT-MIRs) has several advantages and limitations. For instance, FT-NIRs has inexpensive components due to low-cost materials such as glass and quartz compared to FT-MIRs. FT-NIRs also use more robust components, and it is easier to manufacture rugged instruments, involving no moving parts. FT-MIRs in contrast contain more spectral information due to the higher resolution of the fundamental vibrational absorption bands and can identify very complex or similar structures compared to the broad overtone and combination absorption bands in the NIR region (Socaciu et al., 2009; Manley, 2014; Shi and Yu, 2017). Another advantage of FT-MIRs includes fundamental vibrations of molecular bonds within a sample that occur in the “fingerprint” region, making the spectral profiles very sensitive; even very similar molecules can produce quite distinct spectral bands. Compared to FT-NIRs, the absorption bands of the spectra are very broad and overlapped as a result of many chemically different samples which give rise to almost indistinguishable spectral profiles. A detailed description of their advantages and limitations has been reviewed by Arendse et al. (2020).

FT-IR spectroscopy has been successfully used to classify geographical locations to classify geographical sources of oils (Lin et al., 2012) and detect adulteration in a variety of oil products (Yang et al., 2005; Gurdeniz and Ozen, 2009). Several studies have highlighted the application of IR spectroscopy for varying analytical quality attributes evaluation for a variety of oil products. Some of the major attributes accessed using IR spectroscopy include phenolic content, carotenoid content, peroxide value, refractive index, yellowness index, and fatty acids composition. These have been carried out for different oil products like olive oil (Inarejos-García et al., 2013; Cayuela and García, 2017), palm oil (Mba et al., 2014), maize oil (Kahrıman et al., 2019) and vegetable oil (Pereira et al., 2008).

Considering that the chemical composition of pomegranate oil may differ depending on the cultivar or growing region. To our knowledge, limited studies for the application of both FT-NIRs and FT-MIRs for evaluating quality attributes of pomegranate kernel oil, but also limited studies involved testing the robustness of PLS calibration models. The robustness of calibration models has become a critical issue in the application of vibrational spectroscopic techniques and an active area of research (Nicolaï et al., 2007; Magwaza et al., 2014). Our study attempts to evaluate the effects of cultivar differences on the robustness of calibration models and the ability of both FT-NIRs and FT-MIRs to qualitatively classify pomegranate oil based on different cultivars. The development of methods that combines FT-IR spectroscopy and chemometrics has the potential of providing novel input into non-destructive oil quality prediction for both authentication and adulteration application. Therefore, this study is aimed at investigating the feasibility of Fourier transform near-infrared and mid-infrared spectroscopy in evaluating pomegranate kernel oil quality both qualitatively [using principal component analysis (PCA) and orthogonal partial least squares discriminant analysis (OPLS-DA)] and quantitatively [*via* partial least squares regression (PLSr)]. However, very few studies on Vis/NIRS applications in fruit.

## Materials and Methods

### Fruit Supply and Processing

Three different pomegranate cultivars (cv. Wonderful, Acco, Herskawitz) were procured from Sonlia pack-house, Wellington, Western Cape region. A total of 180 fruit or 60 fruit per cultivar was used for this study. At the research laboratory, fruit without any physical defects was sorted and manually cut open for the edible aril portion at ambient conditions (21°C ± 65% RH). Cheesecloth was employed to separate kernels from the arils. Kernels were extracted from arils and then washed with distilled water to eliminate the residual aril sacs before being dried at a temperature of 60°C for 24 h in a hot air oven (PROLAB, South Africa). Pomegranate kernels were dried to a moisture content of 1.7 wt. % (dry basis). After drying the seed the final seed weight averaged 12 ± 2.5 g per fruit. Dried pomegranate kernels were then packed in a polyethylene bag and stored at −20°C until further processing.

### Oil Extraction and Yield

In this study, pomegranate oil was extracted using the solvent extraction method as described by Ampem (2017). Dried kernels were grinded into a powder with a particle size of 0.25 mm using a Sunbeam coffee grinder (Model SCG-250, 60 g capacity, South Africa) in preparation for oil extraction (Eikani et al., 2012). Hexane solvent was used to extract oil from the kernel powder. Pomegranate kernel powder (30 g) was weighed into a glass flask and extracted twice, respectively, with 300 ml of hexane solvent at a time, reaching a total volume of 600 ml solvent solution for each sample. The mixture (600 ml) was sonicated in an ultrasonic bath (Model DC 400H, Haifa, Israel) which was operated at 40°C for 40 min. The oil filtrates from repeated extractions were pooled and recovered through distillation using a rotary evaporator (Heidolph Instruments GmbH & Co. KG, Germany). Thereafter, samples were placed within a vacuum oven at 60°C for 1.5 h to remove any remaining hexane solution (Parashar et al., 2009). A total of 6 ml oil was obtained from each fruit and transferred into a 9 ml glass tube and stored in a dark environment at room temperature until further analysis. A total of 45 oil samples composed of 15 samples each from three different cultivars (Acco, Wonderful, Herskawitz) were used for this study.

### Spectral Acquisition

The Alpha-P ATR FT-IR spectrometer (Bruker Optics, Ettlingen, Germany) and the Multi-purpose analyser (MPA) spectrometer (MPA, Bruker Optics, Ettlingen, Germany) were used for spectral data acquisition. Samples were kept in 8 mm glass vials, and sample temperature was maintained at ±50°C using a heating block before spectra recording. This was to ensure that sample temperature was stable as studies have shown temperature to impact the intensity of the bands (Jiang et al., 2008; Cayuela and García, 2017; Özdemir et al., 2018). The temperature of 50°C was chosen through preliminary trials and consultation with Bruker Optics, South Africa. For the MPA spectrometer, the spectral data were acquired over the range of 12,500 to 4,000 cm^−1^ (scanning resolution 4 cm^−1^; scanner frequency 10 kHz; background with air, 128 scans). The spectral acquisition occurred almost immediately for the Multi-purpose analyser (MPA) spectrometer since the instrument does not have a temperature control system. For the Alpha-P ATR FT-IR spectrometer, sample spectral data were acquired over the range of 4,000–400 cm^−1^. The Alpha-P spectrometer was equipped with a diamond crystal plate (area 2 mm^2^) that maintained the sample temperature at 50°C. The temperature was monitored using OPUS software and spectral acquisition would only occur when the diamond crystal plate and sample reached a temperature of 50°C. The average time taken to acquire spectral data for one sample was 120 s using the following instrument settings: 4 cm^−1^ resolution scan, 10 kHz scanner frequency and 128 averaged scans per spectrum. The sample stage was cleaned in-between measurements with soft paper and undiluted methanol to avoid cross-contamination (Foudjo et al., 2013).

### Reference Measurements

#### Refractive Index

The refractive index of pomegranate oil was measured at ambient temperature (21 ± 3°C) with a calibrated Abbé refractometer, Model 302 (ATAGO Co. Ltd., Japan). Three drops of pomegranate oil were loaded onto the refractometer prism, and refractive index values were reported as mean ± standard error (SE, *n* = 3) for each sample. After each measurement, the prism was cleaned with petroleum ether followed by distilled H_2_O and dried with tissue paper.

#### Yellowness Index

Yellowness index indicates the degree of yellowness associated with scorching, soiling, and general product degradation by light, chemical exposure, and processing. The yellowness index of pomegranate oil was evaluated based on the CIE L*a*b* coordinates from a calibrated Minolta Chroma Meter, Model CR-400 (Japan). The yellowness index was calculated as described by Pathare et al. (2013).


(1) $$YI=\frac{142.86*b^{*}}{L^{*}}$$

#### Total Phenolic Content

Total phenolic content was measured using the Folin–Ciocalteau (Folin C) assay as reported by Makkar et al. (2007) with modification, according to Fawole et al. (2012). Briefly, pomegranate oil (0.5 ml) was dissolved in 14.5 ml of 50% aqueous methanol. An aliquot of 50 μl was diluted with 450 μl of 50% methanol (v/v) before the addition of 1 N Folin C (500 ml) and 2% sodium carbonate (2.5 ml). The mixture was vortexed and stored in a dark environment for 30 min before the absorbance was recorded at 760 nm against blank aqueous methanol. The total phenolic content of pomegranate kernel oil was extrapolated and reported as milligram gallic acid equivalent (mg GAE/g oil). The results for each sample were presented as mean ± SE (*n* = 3).

#### Total Carotenoid Content

Total carotenoid content was evaluated as decribed by Biehler et al. (2010) and Siano et al. (2015) with modification. In brief, pomegranate oil (0.1 ml) was dissolved in 10 ml dimethyl sulfoxide (DMSO). The total carotenoid content of the resulting mixture was recorded at 440 nm and 460 nm, against a blank DMSO solvent. A standard curve consisting of 0.02–0.15 mg/ml DMSO solution was prepared following the same procedure. The results for total carotenoid content were expressed as gram (g β-carotene/kg) of pomegranate oil, and the results for each sample were presented as mean ± SE (*n* = 3).

#### Peroxide Value

Peroxide value was performed as described by Ampem (2017). Briefly, pomegranate oil (0.2 ml) was dissolved in 9 ml of chloroform: methanol mixture (7:3 ratio) in screw-capped vials. The resultant solution was mixed with 50 μl of 10 Mm xylenol orange methanol solution and 50 μl of 36 Mm iron (II) chloride solution and vortexed, respectively. The peroxide value of the resulting mixture was estimated following absorbance reading at 560 nm. Peroxide value was expressed in milli-equivalents (meq) of active oxygen per kilogram of oil and calculated using the following equation:


(2) $$PV=\frac{\left(A_{S}-A_{B}\right)\times mi}{W\times 55.84\times 2}$$

Where

*PV* = peroxide value.
*A_B_* = absorbance of the blank.
*A_S_* = absorbance of the sample.
*mi* = the inverse of the slope (Obtained from calibration curve).
*W* = weight of the sample (g). 55.84 is the atomic weight of iron.

### Chemicals and Reagents

All chemical reagents were obtained from Sigma–Aldrich–Fluka Co. Ltd. (South Africa) unless otherwise stated.

### Chemometric Data Analysis

The spectral acquisition occurred with OPUS software (version 7.0), while data processing and analysis were achieved with SIMCA and OPUS software. Qualitative analysis (modeling of cultivar difference) was carried out using PCA and OPLS-DA using SIMCA software, and quantitative analysis (developing calibration models) was carried out with PLSr using OPUS software. For this study, several preprocessing methods were evaluated, baseline correction spectra were subjected to several filtering techniques, which included Savitzky–Golay transformation (first derivative), multiplicative scattering correction (MSC), and standard normal variate (SNV) correction. Separate OPLS-DA models were built for both NIR and MIR spectral data, each pair of two successive stages by using a dummy variable with a value of 1 assigned to samples that belonged to a specific group and a value of 0 to samples that did not belong to that group.

#### Partial Least Square (PLS) Regression Analysis of Spectral Data

For the quantitative analysis of spectral data, the spectral parameters used for multivariate analysis were optimized by subjecting spectral data to the software’s “Optimise” function. This function provides a combination of parameters such as different pre-processing methods and wavenumber regions and ranks results based on the number of latent variables and root-mean-square error of cross-validation (RMSECV) values.

The development of calibration models for the infrared (NIR and MIR) spectra was performed by applying partial least squares regression analysis (including mean centering). Spectral outliers were identified as having high residual variance from the zero line. Concentration outliers present in the dataset were removed and successive rounds of PLSr were done with the reduced dataset. A total of three outliers were removed, and the resultant calibration models were validated with the test dataset. For PLSr analysis, cross-validation was applied by the Leave-one-out method, which calculates potential models excluding one observation at a time. Calibration models was developed by combining all three cultivars and then randomly splitting the dataset into 2:1 subsets, i.e., calibration (70%) and prediction (30%) sets, each subset containing sufficient samples of each cultivar.

The performance of PLS models was evaluated according to the following prediction statistics: coefficient of determination [*R*^2^; Eq. (3)], root mean square error of validation [RMSEV; Eq. (4)] and root mean square error of prediction [RMSEP; Eq. (5)]. Other statistical indicators for this study include models bias [Eq. (6); which gives an indication of the systematic error in the predicted values and its calculated values] and the residual prediction deviation [RPD; Eq. (7)] value, which is defined as the ratio of the standard deviation of the reference data of the validation set to the RMSEP value (which indicates the efficiency of calibration models). RPD values can be used to evaluate the performance of the developed models (Williams, 2014). According to Nicolaï et al. (2007), models with RPD values below 1.5 is unreliable, while values between 1.5 and 2.0 indicate models can be used for rough prediction, while RPD values between 2.0 and 2.5 can be used for quantitative predictions, any values above 3 are considered satisfactory. The best-performing models were selected based on the best overall performance (low RMSEP, low RMSEV, high *R*^2^, and higher RPD, and low bias).


(3) $$R^{2}=1-\frac{\sum {\left(y_{cal}-y_{act}\right)}^{2}}{\sum {\left(y_{cal}-y_{mean}\right)}^{2}}$$

(4) $$RMSEE=\sqrt{\frac{1}{M-R-1}\times SSE}$$

(5) $$RMSEP=\sqrt{\sum \frac{{\left(y_{pred}-y_{act}\right)}^{2}}{n}}$$

(6) $$Bias=\frac{1}{n}\sqrt{\sum {\left(y_{pred}-y_{act}\right)}^{2}}$$

(7) $$RPD=\frac{SD}{RMSEP}$$

Where *n* is number of spectra, *y*_act_ is actual value, *y*_mean_ is mean value, *y*_cal_ is calculated value, *y*_pred_ is the predicted value of the attribute, *M* is the number of calibration samples, *R* is the rank, SSE is the sum of squared error, SD is the standard deviation of reference values.

### Statistical Analysis

To demonstrate that the prediction of the different selected quality parameters is from the actual IR spectra and not due to possible correlations with the other measured parameters, the reference data was subjected to Pearson’s correlation test using Statistica software (Statistica 16.0, StatSoft Inc., Tulsa, OK, United States).

## Results and Discussion

### Distribution of Calibration and Validation Reference Data

For this study, reference data for the different parameters were normally distributed around the mean (Table 1). According to Lu et al. (2006), the accuracy and validation of calibration models normally depend on large variation in the present within the sample set in the physical and biochemical reference data. However, reports have indicated superior model accuracies using NIR spectroscopy when data with a large sample variation within the calibration and validation set is being considered (Magwaza et al., 2013, 2014). Table 2 presents the standard deviation, minimum-to-maximum range, and CV% statistics of most of the parameters. Most parameters had high CV% values of up to 43% for both calibration and validation data sets covering a wide range of values, aside from the refractive index. Pearson correlation was applied to investigate the interrelationships between selected reference data of pomegranate oil. From the result, it can be deduced that the prediction of these quality parameters (phenolics, carotenoids), and their concentrations should not correlate with one another. Correlation tests indicate that no correlation was observed between chemical indices such as phenolic and carotenoid content (0.227). Similarly, phenolic and carotenoid content showed no correlation with peroxide value (−0.009, 0.334) refractive index (0.260, 0.176) or oil yellowness index (0.172, 0.215). These results suggest that the prediction of the different studied parameters is actually from the IR spectra.

**Table 1:** Mean, standard deviation (SD), range, and coefficient of variation (CV) for calibration and validation subsets for selected parameters of pomegranate kernel oil (sample number = 42).

Parameters	Calibration set				Validation set				Overall CV%
	Mean	SD	Min	Max	Mean	SD	Min	Max	
Peroxide value	7.311	4.219	1.745	16.342	7.478	3.873	1.943	13.517	54.758
Refractive index	1.520	0.0008	1.517	1.523	1.521	0.0010	1.519	1.522	0.0628
Total carotenoid content (g β-carotene/kg)	0.0977	0.0418	0.0640	0.270	0.100	0.0436	0.0650	0.232	43.158
Total phenolic content (mg GAE/g oil)	3.987	0.745	3.113	5.221	3.702	0.436	3.223	4.343	15.247
Yellowness index	54.226	18.540	23.141	97.280	55.690	21.842	23.946	96.416	36.706

**Table 2:** Model evaluation statistics for quality parameters of pomegranate kernel oil as determined from FT-NIR and FT-MIR spectroscopy (sample number = 42).

Parameter	Acquisition mode	Pre-processing	Wavenumbers range (cm^−1^)		Calibration		Validation					
				LV	*R* ^2^	RMSEV	*R* ^2^	RMSEP	RPD	Bias	Slope	Corr.
Peroxide value	FT-NIRs	1st + MSC	7,500–6,098, 5,450–4,597	3	0.833	1.68	0.833	1.78	2.80	−0.866	0.823	0.935
	FT-MIRs	2nd	3,996–2,556	3	0.959	0.99	0.919	1.05	3.54	−0.036	0.829	0.965
Refractive index	FT-NIRs	2nd	9,400–6,098, 5,450–4,597	4	0.904	0.0003	0.863	0.0003	3.44	−0.000	0.906	0.956
	FT-MIRs	2nd	3,996–1,118	4	0.960	0.0002	0.912	0.0002	3.43	0.000	0.867	0.958
Total carotenoid content	FT-NIRs	SLS	7,498–940, 6,102–5,774	5	0.892	0.015	0.843	0.019	2.28	−0.003	0.893	0.944
	FT-MIRs	2nd	3,996–3,635, 2,558–1837, 760–399	3	0.958	0.002	0.632	0.007	1.72	0.002	0.543	0.836
Total phenolic content	FT-NIRs	SLS	7,502–4,597	2	0.332	0.85	0.185	1.39	1.26	0.657	0.226	0.774
	FT-MIRs	1st	3,996–3,965, 1,479–758	2	0.635	0.47	0.568	0.37	1.57	−0.066	0.879	0.814
Yellowness index	FT-NIRs	2nd	8,451–7,498, 6,102–4,597	5	0.556	13.60	0.531	14.30	1.49	2.64	0.543	0.740
	FT-MIRs	2nd	2,918–2,556, 1,120–758	1	0.307	11.90	0.205	15.00	1.15	−3.10	0.267	0.491

**R*^2^, coefficient of determination; RMSEV, root mean square error of validation; RMSEP, root mean square error of prediction; RPD, residual predictive deviation; LV, latent variable; Corr., correlation coefficient; 1st, first derivative; 2nd, second derivative; MSC, multiplicative scattering correction; SLS, straight line subtraction*.

### FT-NIR and FT-MIR Spectral Characteristics of Pomegranate Oil

**Figure 1:**
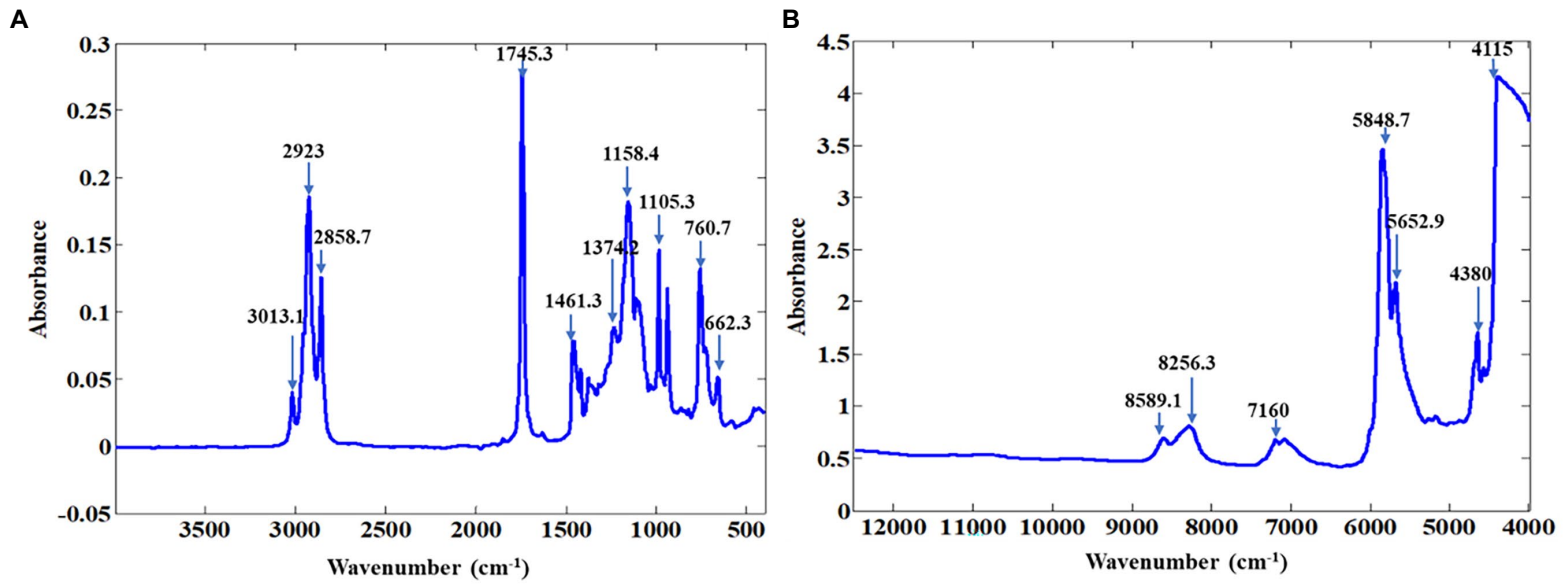
Representative absorbance spectra for ATR-FT-MIR **(A)** and FT-NIR **(B)** of pomegranate kernel oil.

NIR spectroscopy is a powerful non-destructive technique used for the detection of various compounds, the NIR spectrum provides information on the vibrational absorption of hydroxyl (O–H), amido (N–H), and C–H bonds. The average for both NIR and MIR spectra of pomegranate oil is presented in Figure 1. Pomegranate is highly abundant in punicic acid (C18:3-9c), linoleic acid (C18:2), and oleic acid (C18:1). Band assignment was done according to the literature (Foudjo et al., 2013; Inarejos-García et al., 2013; Özdemir et al., 2018). In the NIR region, bands around 8,451 cm^−1^ arise from second overtones of C–H stretching vibrations, while those at 7,502 and 7,498 cm^−1^ are due to the combination bands of C–H in fatty acids. The bands at 5,774 and 5,450 cm^−1^ can be ascribed, according to literature, from the first overtone of C–H stretching vibrations of methyl, methylene, and ethylene groups (Sinelli et al., 2011; Özdemir et al., 2018). Small bands at 4,659 and 4,597 cm^−1^ are associated with combination bands of C–H and C–O stretching vibration. Several bands dominate the MIR spectra at 2,918, 2,556, 1,837, 1,463, 1,377, 1,238, 1,163, 1,114, 1,099, and 721 cm^−1^. The absorbance band at 3,013 cm^−1^ has been associated with the stretching of the functional group =C-H (cis-) found in unsaturated fatty acids such as punicic acid (Guillén and Cabo, 1997). Absorbance at 2,924 and 2,852 cm^−1^ are due to bands from asymmetric CH_2_ stretching vibration of acyl chains and methylene chains in fatty acids (punicic acid, linoleic acid, and oleic acid; Guillén and Cabo, 1997; Sun, 2009). The major band at 1,743 cm^−1^ arises from C=O stretching vibrations of ν(C=O) ester in fatty acids (Guillén and Cabo, 1997, Sun, 2009). The band at 1,238, 1,163 and 1,114 cm^−1^ has been associated with C-O or CH_2_ stretching or bending vibration out-of-plane of functional groups from fatty acids (Guillén and Cabo, 1997; Rohman and Che, 2011). The band at 721 cm^−1^ corresponds to the CH_2_ rocking mode (Yang et al., 2005; Sinelli et al., 2010). The spectral profile for pomegranate oil is comparable to those reported for other oil samples like avocado oil (Foudjo et al., 2013), virgin olive oil (Dupuy et al., 2010; Sinelli et al., 2010), rapeseed oil blend (Ma et al., 2014), and palm oil (Mba et al., 2014).

### Qualitative Analysis of Pomegranate Oil Using PCA and OPLS-DA

#### Unsupervised Clustering (PCA)

PCA is, arguably, one of the most useful and widespread unsupervised methods used in chemometrics for its exploratory data analyses (Cozzolino et al., 2011; Jolliffe and Cadima, 2016). PCA was carried out to explore the possible clustering of samples and evaluate the influence of cultivar on oil quality. PCA is a statistical technique that is used to investigate the structure of a data set and attempts to model the total variance of the original data set *via* the uncorrelated principal components (Filzmoser and Todorov, 2011; Gautam et al., 2015). PCA maximizes the variation in the data set projects the main variation onto a few latent variables and presents sample groupings as clusters in PCA score plots with the corresponding loadings plots (Wold et al., 1987).

**Figure 2:**
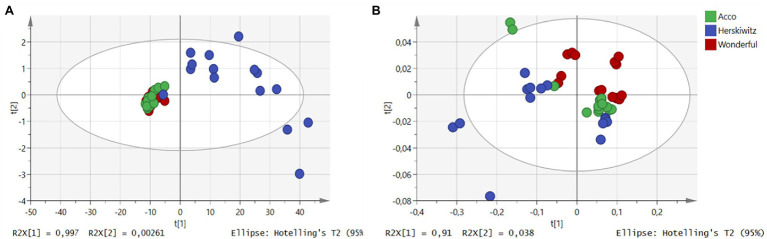
PCA score plots for NIR spectral data **(A)** and MIR spectral data **(B)**. The colour represents different cultivars of extracted pomegranate oil, green (Acco), blue (Herskawitz), and red (Wonderful) (sample number = 42).

Preliminary assessment of both NIR and MIR spectra was performed using PCA, to examine the effects of cultivar differences on pomegranate oil quality. For NIR baseline-corrected spectra, the first two principal components (PC) were used in relation to the chemical variation within the sample sets (Supplementary Table 1). By plotting all 45 data points, scores from the first two PC explained 100% (PC_1_ = 99.97%, PC_2_ = 0.0026%) of the total variation within the dataset. One dispersed group (cv. Herskawitz) was observed, which revealed that the first PC contributed most to the sample distribution, where samples were mainly stretched along the PC_1_ region (Figure 2A). However, the PCA plot revealed no clear groupings according to chemical variation within the data set, with cultivar Acco co-clustering with cultivar Wonderful. The score plots from the FT-MIR spectra for oil samples showed that the first two PC explained a total of 91.38% (PC_1_ = 91%, PC_2_ = 0.38%) of the variation (Supplementary Table 2). Examination of the PCA scores plot generated from three cultivars showed well-defined sample clusters for both Acco and Wonderful cultivars with both co-clustered with cv. Herskawitz (Figure 2B). This observation showed that despite its simplified approach, IR spectroscopy could be used to differentiate between different cultivars based on spectral data.

#### Supervised Clustering/Discriminant Analysis (OPLS-DA)

Orthogonal projections to latent structures discriminant analysis (OPLS-DA) is a supervised classification technique that isolates a predictive component and integrates an orthogonal correction filter, to differentiate the variation within the dataset (Bylesjö et al., 2006). OPLS-DA is often used as an alternative method, where PCA cannot show clear clustering. OPLS-DA works through the projection of data and is guided by known class information, thus offering increased separation projection in comparison to PCA (Trygg et al., 2007). This is because OPLS-DA score plots are rotated so that between-class variation is projected on the predictive component, while within-class variation, is projected on the first y-orthogonal component (Wiklund et al., 2008). Therefore, several authors classify that OPLS-DA models are easier to interpret than PLS-DA models, although both methods have the same predictive power (Trygg and Wold, 2002; Trygg et al., 2007; Musingarabwi et al., 2016).

**Figure 3:**
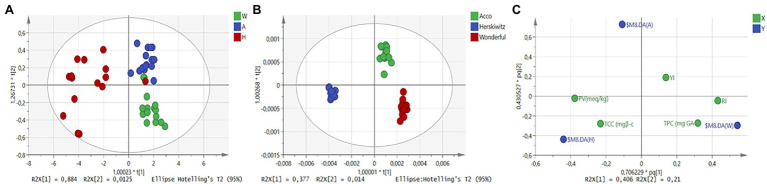
OPLS-DA scores plots for NIR baseline-corrected spectra **(A)**, MIR baseline-corrected spectra **(B)**, and reference data plot **(C)**. The colour in **(A)** and **(B)** represents different cultivars (Acco, Wonderful, Herskawitz) of extracted pomegranate oil (sample number = 42).

To see the effects of cultivar differences on the quality characteristics of pomegranate oil, OPLS-DA was performed on both FT-NIR and FT-MIR spectra (Figure 3). For FT-NIRs, the application of OPLS-DA showed two well-clustered groups (cv. Wonderful and Acco), while pomegranate cultivar Herskawitz remained dispersed and co-clustered with cultivar Wonderful (Figure 3A). Whereas, for FT-MIRs, the application of OPLS-DA successfully discriminated and separated all three cultivars into well-defined cluster groups (Figure 3B). A similar approach to qualitatively evaluate the quality of grape berries was performed by Musingarabwi et al. (2016). These authors reported that the application of PCA and OPLS-DA successfully discriminated against and separated different developmental stages of grape berries into well-defined cluster groups. The ability to successfully discriminate between different cultivars by both PCA and OPLS-DA using IR spectra has the potential for the application of authentication and adulteration assessment of pomegranate oil.

Interestingly, when plotting the reference data for each cultivar that is based on the reference measurements, the cultivar Herskawitz was highly associated with peroxide value and total carotenoid content (Figure 3C). The Wonderful cultivar has been grouped with total phenolic content and refractive index. While cv. Acco has shown an association with the yellowness index. These results suggest that the Wonderful cultivar has the highest concentration of unsaturation fatty acids since fatty acids are directly proportional to the refractive index of the oil. Similarly, pomegranate (cv. Wonderful) has the highest phenolic content, suggesting that fruit consumption for this particular cultivar, increases the intake of phenolic compounds which have been linked to antioxidant compounds. For color attributes, pomegranate oil obtained from Acco cultivar was the most suitable to assess the characteristic yellow coloration. While the cultivar Herskawitz has a high peroxide value suggesting that oil obtained from Herskawitz cultivar is more susceptible to oxidation. The differentiation between cultivars may be due to cultivar differences or fruit maturity status.

### Quantitative Analysis of Pomegranate Oil Using PLS Regression

**Figure 4:**
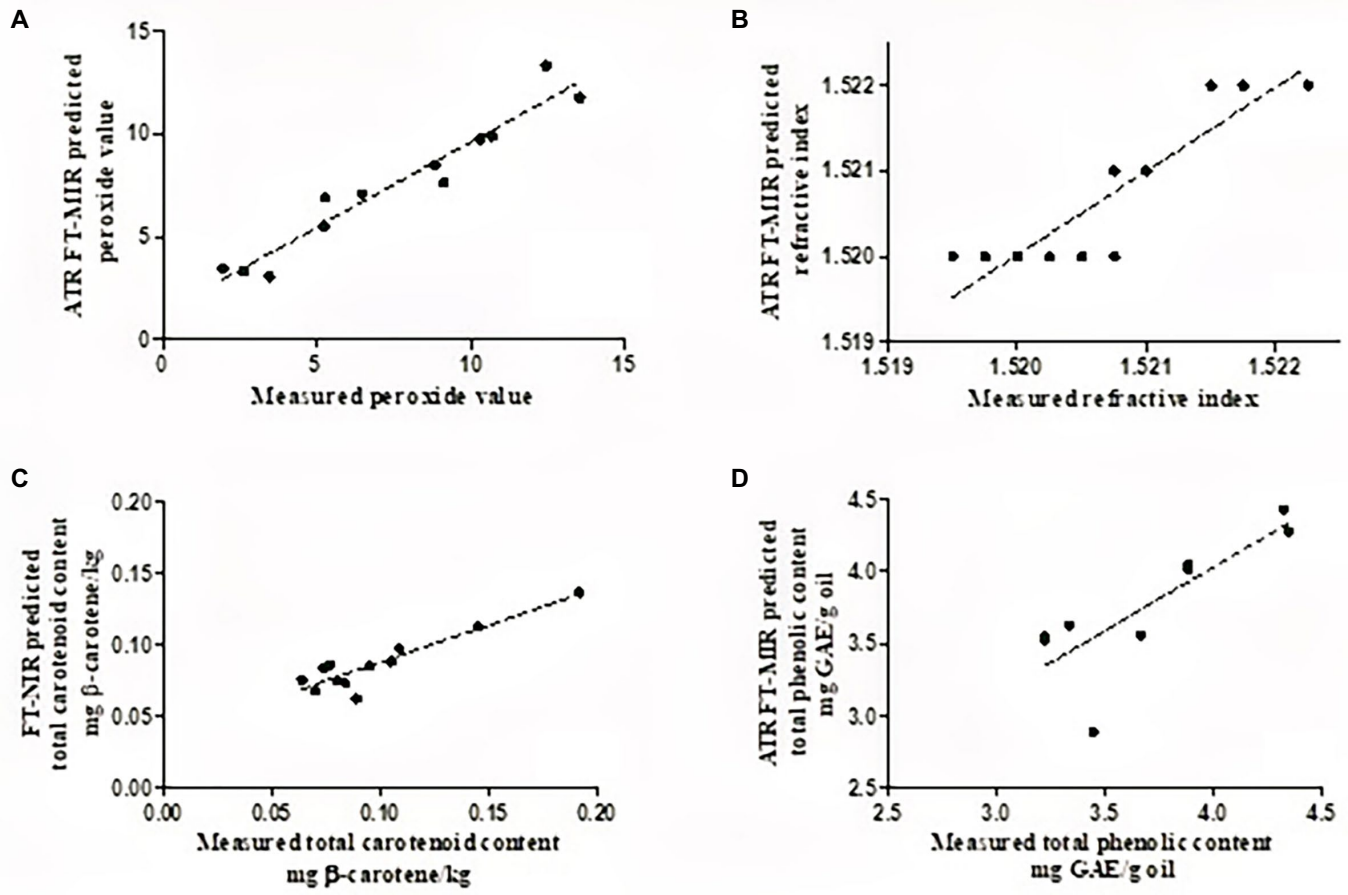
Scatter plots of FT-NIR/FT-MIR predicted **(A)**, refractive index **(B)**, total carotenoid content **(C)**, and total phenolic content **(D)**, plotted against destructively acquired reference data (sample number = 42).

The best FT-NIRs and FT-MIRs models were developed using 17 points, first derivative, second derivative, and straight-line subtraction, respectively. The model for each parameter was selected based on the evaluation of statistical parameters that gave higher *R*^2^, high RPD values, lowest RMSEV and RMSEP, and lowest number of latent variables. The overall performance of the developed models for all quality parameters is represented in Table 2. Scatter plots of FT-NIR and FT-MIR spectroscopy for predicted data plotted against measured reference data are presented in Figure 4. Models developed in the NIR and MIR spectral regions had a major influence on the regression statistics. All three cultivars were combined to create models with high robustness and variability.

The refractive index is an intrinsic property of oil measured based on light penetration through an oil sample (Aydeniz et al., 2014; Khoddami et al., 2014). Oil refractive index has been reported to be directly proportional to the degree of unsaturation of fatty acids and inversely related to its viscosity and can, therefore, be used to quantify the double bonds of fatty acids (Aydeniz et al., 2014; Khoddami et al., 2014). The statistical indicators for model fitness showed that both NIR and MIR spectra yielded relatively accurate PLS models for refractive index. PLS model development in the FT-MIRs provided slightly better prediction statistics (*R*^2^ = 0.912, RMSEP = 0.0002, and RPD = 3.43) compared to the FT-NIRs (*R*^2^ = 0.863, RMSEP = 0.0003, and RPD = 3.44; Table 2). The wavenumber range used during the development of the PLSr model for the refractive index was between 3,996 and 1,118 cm^−1^, which is within the range reported by Yang and Irudayaraj (2001) for olive oil. The RPD value suggests the model developed can provide a satisfactory prediction for refractive index, while the low bias (<0.0002) of the developed model suggests that the model was stable and non-sensitive to factors such as cultivar.

For quality control in edible fats and oils, oxidation is one of the main parameters used for oil products. Oxidation of fats and oils produces either primary (peroxides) or secondary oxidation products. The PLSr models developed for peroxide value has shown that the FT-MIRs provided better prediction statistics (*R*^2^ = 0.919, RMSEP = 1.05 meq, and RPD = 3.54) compared to the FT-NIRs (*R*^2^ = 0.833, RMSEP = 1.78 meq, and RPD = 2.80; Table 2). Similar values were observed for RMSEV and RMSEP, suggesting that the developed models were not overfitted. Furthermore, the developed models had low bias values (0.036–0.86) indicating robust fitting and stability. This indicates that the models were not sensitive to external factors such as different cultivars. The RPD value (3.54) for the developed model suggests that satisfactory predictions can be made with FT-MIRs.

FT-MIR spectroscopy has been used to evaluate the peroxide value of coconut oil, where the authors reported a high coefficient of determination (*R*^2^ = 0.982) and low RMSEP values (0.4978 meq; Marina et al., 2013). The wavenumber range reported in this study for peroxide value is similar to those reported for the development of models for various oil products (Liang et al., 2013; Marina et al., 2015; Zahir et al., 2017). For total carotenoid content, the NIR region of 7,498–940 and 6,102–5,774 cm^−1^ provided better prediction statistics (*R*^2^ = 0.843, RMSEP = 0.019 g β-carotene/kg) compared to FT-MIRs (*R*^2^ = 0.632, RMSEP = 0.007 g β-carotene/kg), with RPD value of 2.28 suggesting that the model is fit for quantitative predictions. Similar prediction results for total carotenoid content were reported by Schulz et al. (1998) in essential oils, within the spectral region of 10,100 and 5,150 cm^−1^. For total phenolic content, FT-MIRs in the region of 3,996 and 758 cm^−1^, have been shown to provide rough predictions (RPD = 1.57), while those developed in the NIR region were shown to be unreliable (RPD = 1.26). Contrary to our results, Trapani et al. (2016) reported that the NIR spectral region of 12,500 to 4,000 cm^−1^ provided relatively good prediction statistics (*R*^2^ = 0.71, RMSEP = 0.08 mg/kg dm) for total phenolic content in olive oil. Model development for the yellowness index gave relatively poor prediction statistics for both FT-NIRs and FT-MIRs (Table 2). Low RPD values and high bias characterized these models, suggesting that the developed models were unreliable, and overestimation may have occurred for these quality attributes. The developed calibration models were only performed using internal cross-validation and thus only applicable to the three selected cultivars. It is well known that the real challenge with calibration models is that their predictive performance almost always reduces when tested on unknown sources such as fruit maturity, seasonality, and growing regions. Thus future research should include more variability (growing regions and seasonality) to improve the model’s robustness.

For this study, FT-MIRs were shown to be better suited for both qualitative and quantitative applications. The regression models developed within the MIR spectral region performed better than those developed within NIR spectral region. This can be because the mid-infrared spectrum contains wavenumbers for fundamental rotational molecular vibration, which is highly sensitive to specific chemical compositions. In contrast, the near-infrared spectrum is associated mainly with overtone and combination bands of fundamental transition, making it less reproducible and specific. Another advantage of ATR FT-MIR spectroscopy is temperature control *via* the ATR crystal, which reduces potential variation by maintaining constant sample temperature (Smyth and Cozzolino, 2011). However, FT-NIRS is more applicable to practical usage for online or inline implementation or the development of portable devices due to their relatively inexpensive instrumentation costs, more robust components, and it is easier to manufacture rugged instruments, involving no moving parts.

## Conclusion

Classification of pomegranate oil quality according to their respective cultivars was possible with FT-IR spectroscopy. FT-MIRs spectra resulted in 100% discrimination between oil samples extracted from different cultivars using OPLS-DA. For quantitative prediction of various quality attributes, FT-MIRs predicted were able to predict three parameters (refractive index, peroxide value, total phenolic content) compared to FT-NIRS (refractive index, total carotenoid content). This study also revealed that pomegranate oil (cv. Wonderful) has been associated with a higher refractive index (indirect correlation with unsaturation fatty acids) and phenolic content compared to “Acco” and “Herskawitz.” This studThe measurement of additional quality characteristics such as individual fatty acids will foreseeably improve the discrimination and prediction accuracy. Future research is required to improve the robustness of calibration models for both NIR and MIR spectroscopy by either increasing the sample size, including different growing locations and seasonality or by applying different chemometric techniques. The current knowledge obtained from this study has shown that chemical indices of pomegranate kernel oil differ even amongst cultivars and are detectable with both FT-NIR and FT-MIR spectroscopy. These chemical indices can not be used for quality evaluation but can be applied to effectively classify or discriminate between oil samples that have even slightly different chemical characteristics, making it a highly effective tool within the processing industry for authenticity and adulteration testing. The approach provides a powerful way to rapidly extract qualitative and quantitative information emanating from multiple spectral variables.

## Data Availability Statement

The original contributions presented in the study are included in the article/Supplementary Material, further inquiries can be directed to the corresponding author.

## Author Contributions

UO: conceptualization, methodology, funding acquisition, project administration, supervision, review, and editing. WP: project administration and supervision. HN: methodology, software and validation, review, and editing. EA: supervision, review, and editing. EO: data curation and writing—original draft preparation. All authors contributed to the article and approved the submitted version.

## Funding

This work is based on the research supported wholly/in part by the National Research Foundation of South Africa (Grant Number: 64813). The opinions, findings, conclusions, or recommendations expressed are those of the author(s) alone, and the NRF accepts no liability whatsoever in this regard.

## Conflict of Interest

The authors declare that the research was conducted in the absence of any commercial or financial relationships that could be construed as a potential conflict of interest.

## Publisher’s Note

All claims expressed in this article are solely those of the authors and do not necessarily represent those of their affiliated organizations, or those of the publisher, the editors and the reviewers. Any product that may be evaluated in this article, or claim that may be made by its manufacturer, is not guaranteed or endorsed by the publisher.
