# Supplementary material for: Non-destructive Evaluation of the Quality Characteristics of Pomegranate Kernel Oil by Fourier Transform Near-Infrared and Mid-Infrared Spectroscopy
Source: Front Plant Sci. 2022 Jul 7;13:867555. doi: 10.3389/fpls.2022.867555 (PMC9301966; doi:10.3389/fpls.2022.867555)
Supplement: Supplementary file 1 [file Data_Sheet_1.docx]

*Supplementary material*

Table 1

PCA model based on NIR spectra for three groups (cv. Wonderful, Acco, Herskiwitz) with overall performance statistics. Observations = 45

| Component | R^2^X | R^2^X(cum) | Eigenvalue | Q^2^ | Limit | Q^2^(cum) | Significance | Iterations |
| --- | --- | --- | --- | --- | --- | --- | --- | --- |
| 1 | 0.997 | 0.997 | 44.9 | 0.996 | 0.0227 | 0.996 | R1 | 3 |
| 2 | 0.001 | 0.999 | 0.117 | 0.773 | 0.232 | 0.999 | R1 | 8 |

R^2^X: the amount of variation in X that is correlated to Y; R^2^X (cum): predicted variation in X that is explained by the model; Eigenvalue: The number of X variables times R^2^X; Q^2^ (cum): goodness of prediction, calculated by full cross-validation; Significance: CV insignificant (NS) or significant according to rule R_z_

Table 2

PCA model based on MIR spectra for three groups (cv. Wonderful, Acco, Herskiwitz) with overall performance statistics. Observations = 44

| Component | R^2^X | R^2^X(cum) | Eigenvalue | Q^2^ | Limit | Q^2^(cum) | Significance | Iterations |
| --- | --- | --- | --- | --- | --- | --- | --- | --- |
| 1 | 0.91 | 0.91 | 40.1 | 0.903 | 0.0233 | 0.903 | R1 | 5 |
| 2 | 0.038 | 0.948 | 0.167 | 0.362 | 0.0238 | 0.938 | R1 | 15 |

R^2^X: the amount of variation in X that is correlated to Y; R^2^X (cum): predicted variation in X that is explained by the model; Eigenvalue: The number of X variables times R^2^X; Q^2^ (cum): goodness of prediction, calculated by full cross-validation; Significance: CV insignificant (NS) or significant according to rule R_z_
